# Supplementary material for: Clustering of susceptible individuals within households can drive measles outbreaks: an individual-based model exploration
Source: Sci Rep. 2020 Nov 12;10:19645. doi: 10.1038/s41598-020-76746-3 (PMC7665185; doi:10.1038/s41598-020-76746-3)
Supplement: Supplementary file 1 — Supplementary Figures. [file 41598_2020_76746_MOESM1_ESM.pdf]

Supplementary Information for: Clustering of susceptible individuals within households can drive measles outbreaks: an individual-based model exploration

Elise Kuylen[1,2,*], Lander Willem[1], Jan Broeckhove[3], Philippe Beutels[1], and Niel Hens[1,4]

[1]Centre for Health Economics Research and Modelling Infectious Diseases (CHERMID), Vaccine and Infectious Disease Institute, University of Antwerp, Antwerp, Belgium
[2]Discipline Group Computer Sciences, Hasselt University, Hasselt, Belgium
[3]IDLab, Department of Mathematics and Computer Science, University of Antwerp, Antwerp, Belgium
[4]I-BioStat, Data Science Institute, Hasselt University, Hasselt, Belgium
[*]elise.kuylen@uantwerpen.be

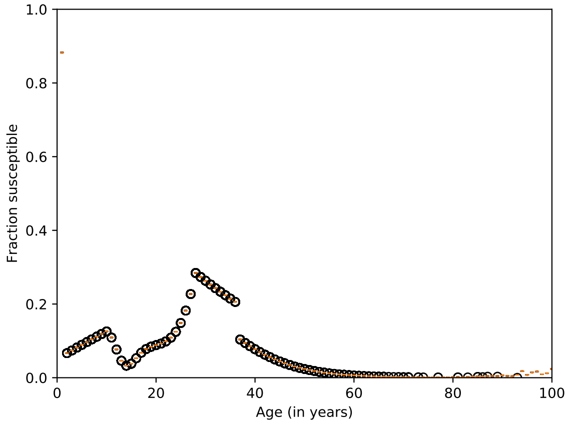

(a) Clustering level = 0.00

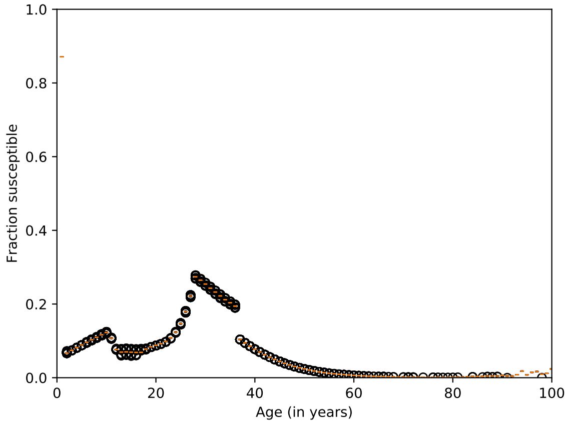

(b) Clustering level = 0.25

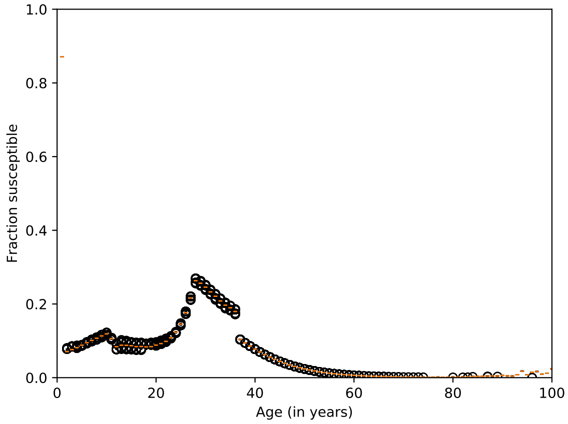

(c) Clustering level = 0.50

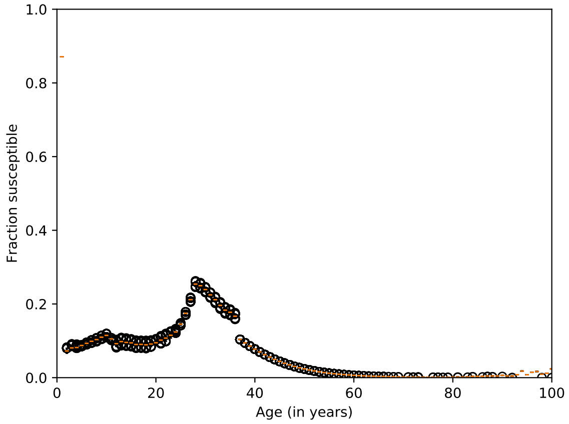

(d) Clustering level = 0.75

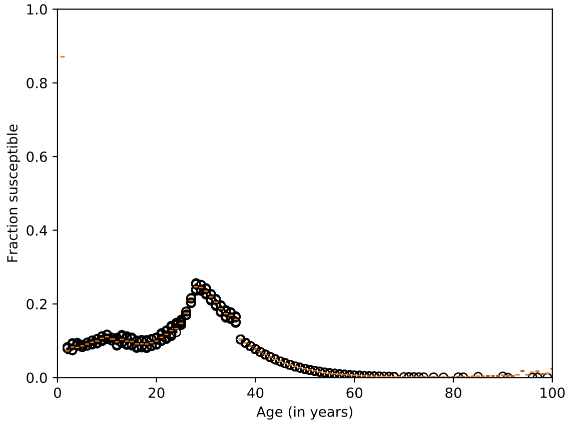

(e) Clustering level = 1.00

Supplementary Figure S1. Box-plots of immunity levels by age for target clustering levels 0.00, 0.25, 0.50, 0.75 and 1.00, each over 13,000 stochastic simulations.

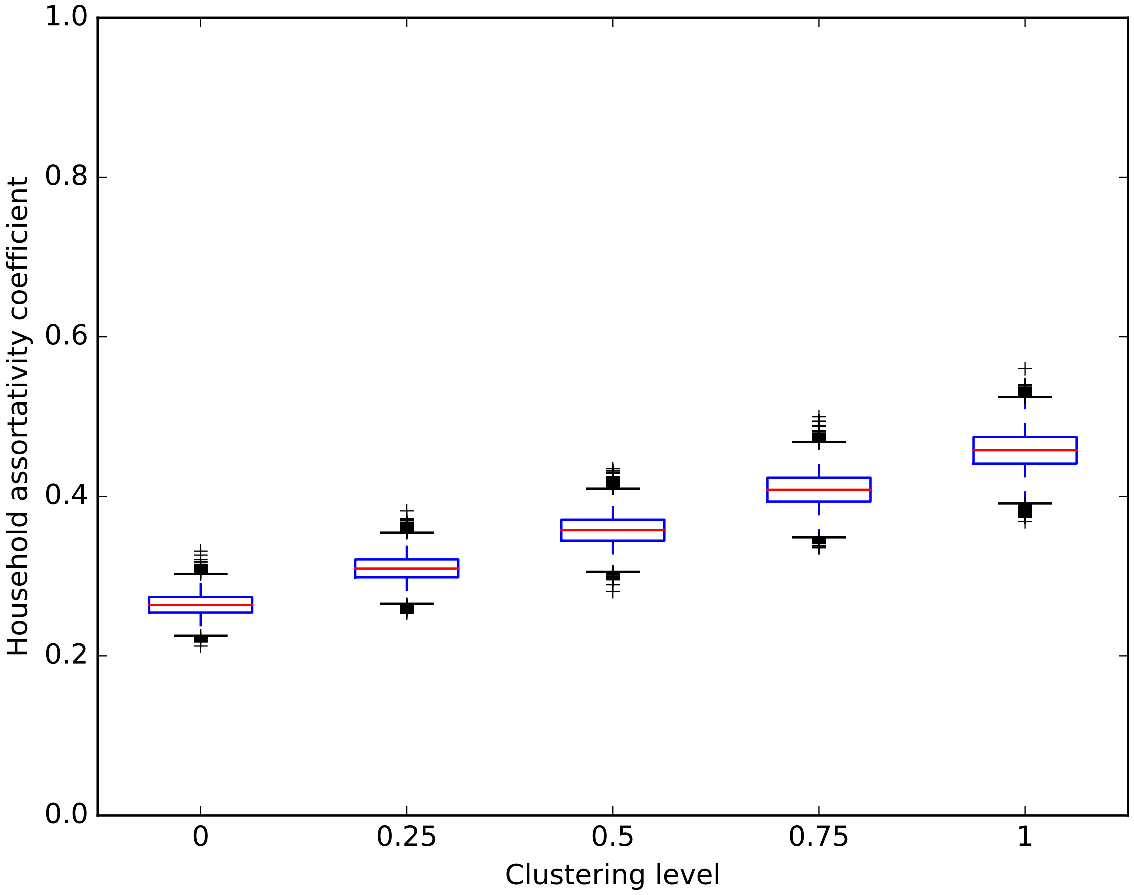

Supplementary Figure S2. Box-plot of household assortativity coefficients based on a sample of 1,000 households, observed for clustering levels 0, 0.25, 0.5, 0.75, 1. Results are based on 13,000 stochastic simulations per clustering level.

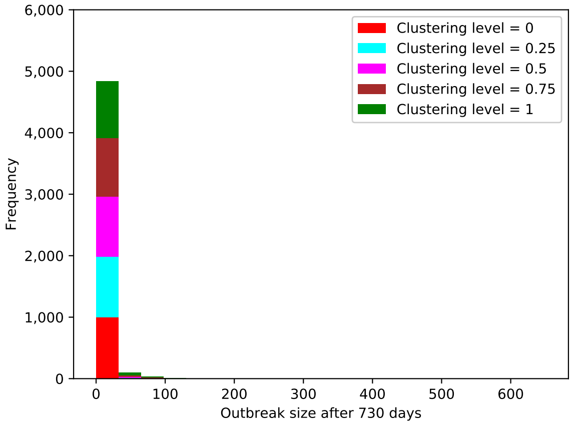

(a) Transmission probability = 0.20 ($\hat{R_0} = 6.80$)

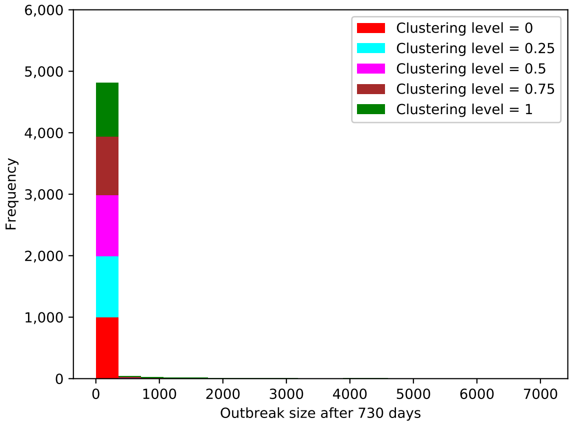

(b) Transmission probability = 0.25 ($\hat{R_0} = 8.38$)

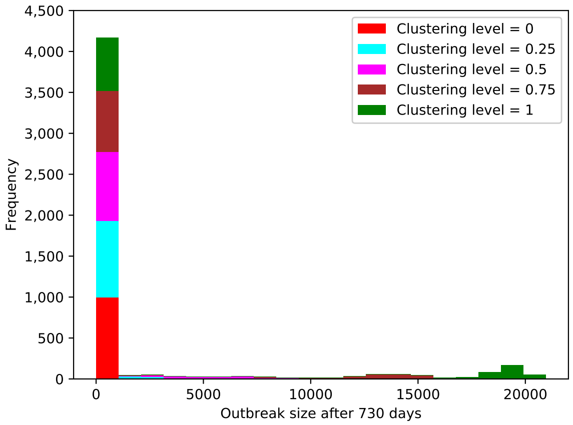

(c) Transmission probability = 0.30 ($\hat{R_0} = 9.91$)

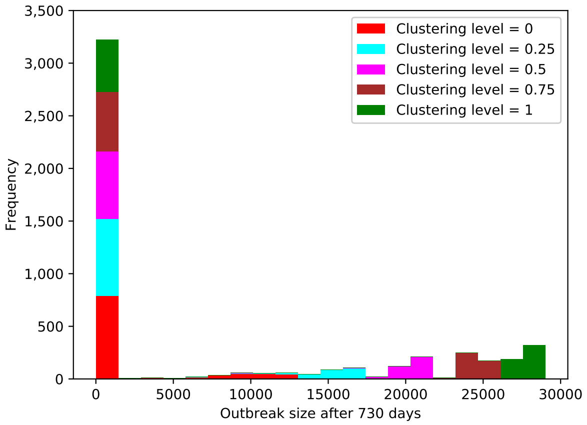

(d) Transmission probability = 0.35 ($\hat{R_0} = 11.38$)

Supplementary Figure S3. Histograms of frequencies of outbreak sizes for clustering levels 0, 0.25, 0.5, 0.75, and 1 over transmission probabilities from 0.20 ($\hat{R_0} = 6.80$) to 0.35 ($\hat{R_0} = 11.38$). Results over 1,000 stochastic simulations per combination of transmission probability and clustering level.

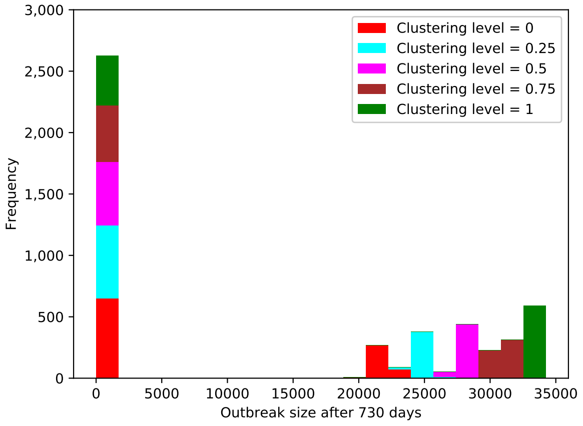

(a) Transmission probability = 0.40 ($\hat{R_0} = 12.80$)

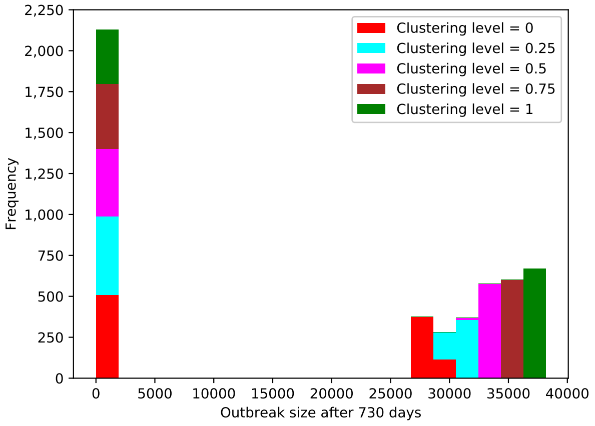

(b) Transmission probability = 0.45 ($\hat{R_0} = 14.16$)

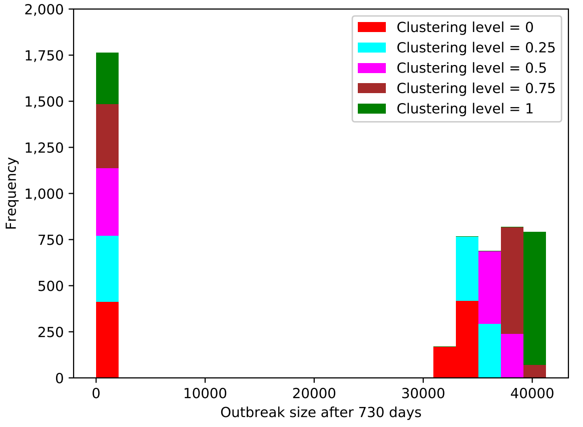

(c) Transmission probability = 0.50 ($\hat{R_0} = 15.48$)

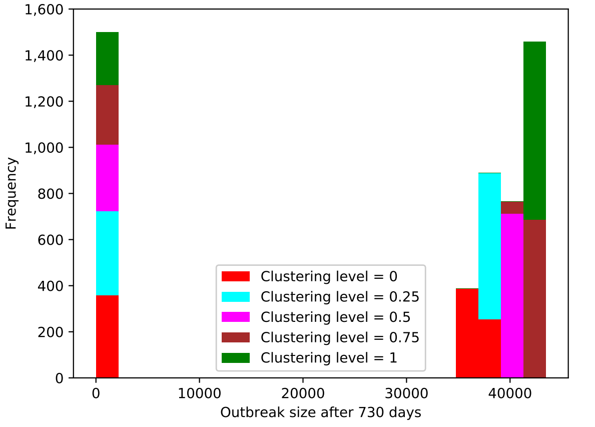

(d) Transmission probability = 0.55 ($\hat{R_0} = 16.76$)

Supplementary Figure S4. Histograms of frequencies of outbreak sizes for clustering levels 0, 0.25, 0.5, 0.75, and 1 over transmission probabilities from 0.40 ($\hat{R_0} = 12.80$) to 0.55 ($\hat{R_0} = 16.76$). Results over 1,000 stochastic simulations per combination of transmission probability and clustering level.

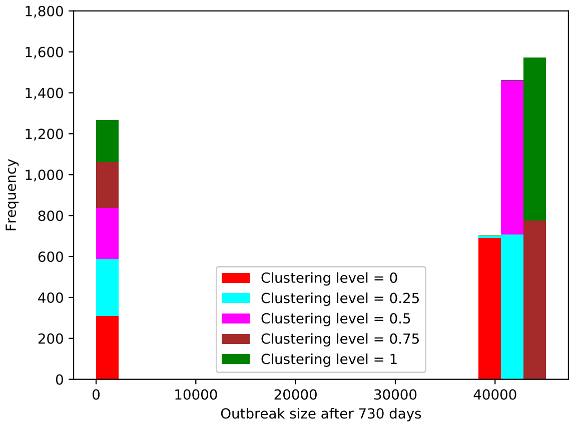

(a) Transmission probability = 0.60 ($\hat{R_0} = 17.99$)

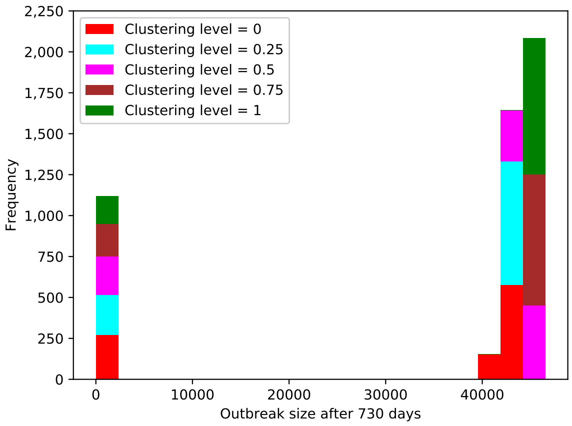

(b) Transmission probability = 0.65 ($\hat{R_0} = 19.19$)

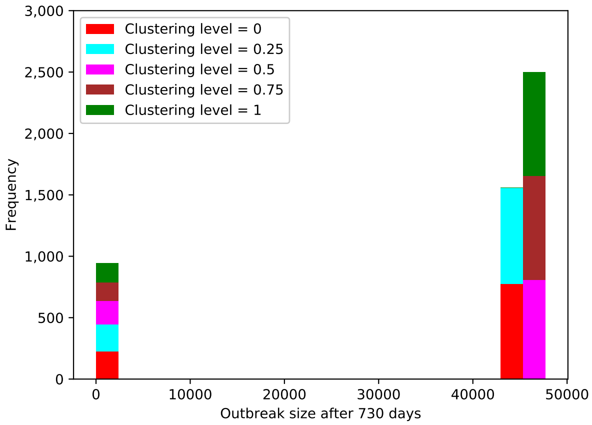

(c) Transmission probability = 0.70 ($\hat{R_0} = 20.35$)

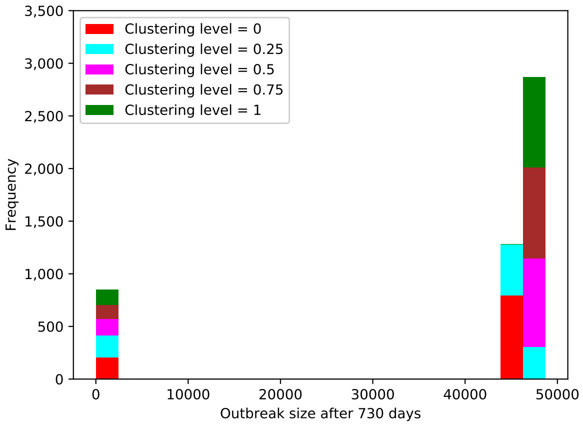

(d) Transmission probability = 0.75 ($\hat{R_0} = 21.48$)

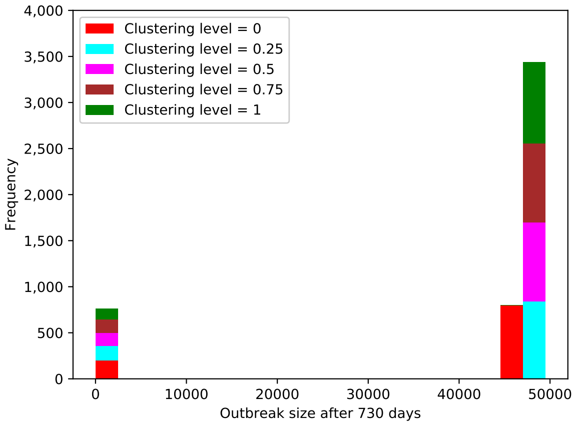

(e) Transmission probability = 0.80 ($\hat{R_0} = 22.58$)

Supplementary Figure S5. Histograms of frequencies of outbreak sizes for clustering levels 0, 0.25, 0.5, 0.75, and 1 over transmission probabilities from 0.60 ($\hat{R_0} = 17.99$) to 0.80 ($\hat{R_0} = 22.58$). Results over 1,000 stochastic simulations per combination of transmission probability and clustering level.

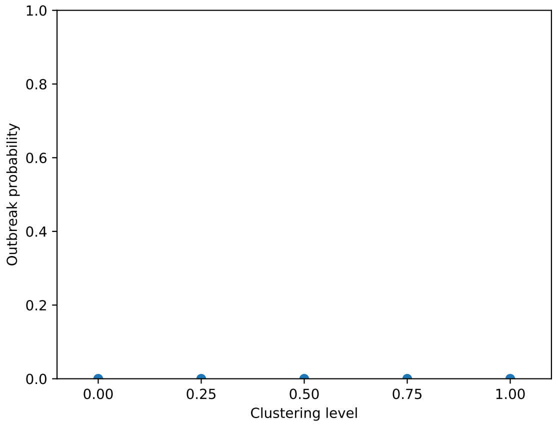

(a) Transmission probability = 0.20 ($\hat{R_0} = 6.80$)

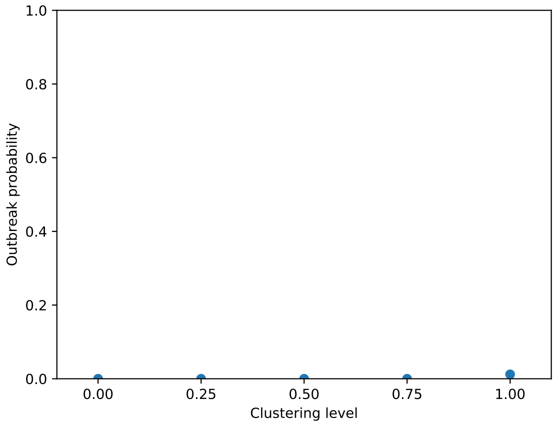

(b) Transmission probability = 0.25 ($\hat{R_0} = 8.38$)

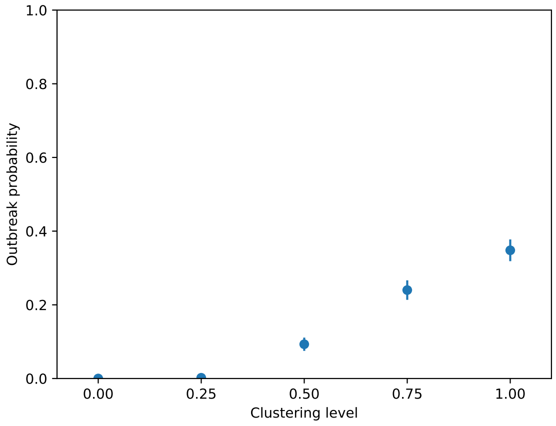

(c) Transmission probability = 0.30 ($\hat{R_0} = 9.91$)

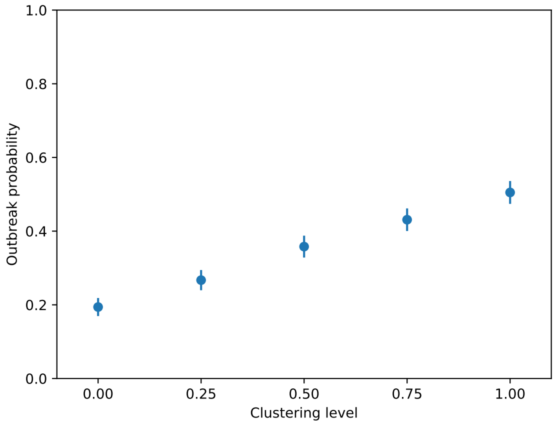

(d) Transmission probability = 0.35 ($\hat{R_0} = 11.38$)

Supplementary Figure S6. Probability of the occurrence of a persistent outbreak for clustering levels 0, 0.25, 0.5, 0.75, and 1 over transmission probabilities from 0.20 ($\hat{R_0} = 6.80$) to 0.35 ($\hat{R_0} = 11.38$). Results over 1,000 stochastic simulations per combination of transmission probability and clustering level. Error bars indicate 95% confidence intervals, for scenarios where enough (at least 5 of each) persistent outbreaks and extinction cases occurred.

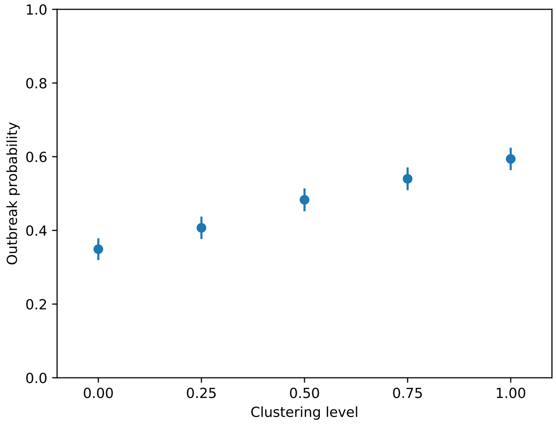

(a) Transmission probability = 0.40 ($\hat{R_0} = 12.80$)

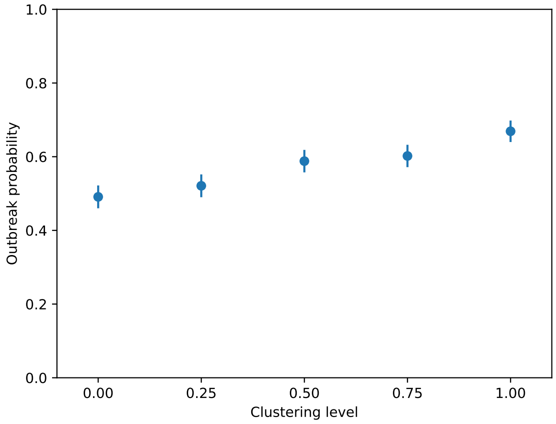

(b) Transmission probability = 0.45 ($\hat{R_0} = 14.16$)

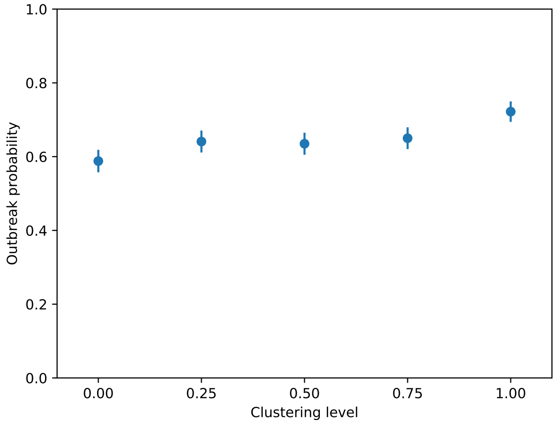

(c) Transmission probability = 0.50 ($\hat{R_0} = 15.48$)

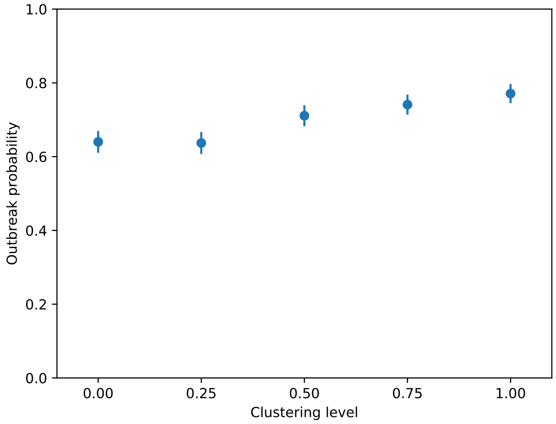

(d) Transmission probability = 0.55 ($\hat{R_0} = 16.76$)

Supplementary Figure S7. Probability of the occurrence of a persistent outbreak for clustering levels 0, 0.25, 0.5, 0.75, and 1 over transmission probabilities from 0.40 ($\hat{R_0} = 12.80$) to 0.55 ($\hat{R_0} = 16.76$). Results over 1,000 stochastic simulations per combination of transmission probability and clustering level. Error bars indicate 95% confidence intervals, for scenarios where enough (at least 5 of each) persistent outbreaks and extinction cases occurred.

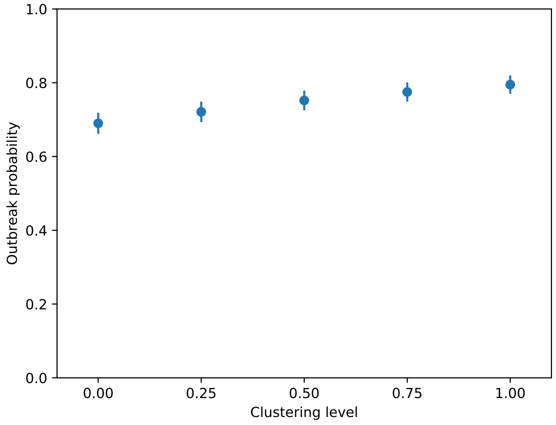

(a) Transmission probability = 0.60 ($\hat{R_0} = 17.99$)

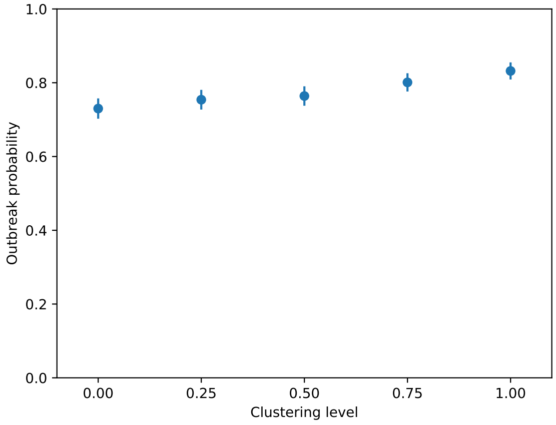

(b) Transmission probability = 0.65 ($\hat{R_0} = 19.19$)

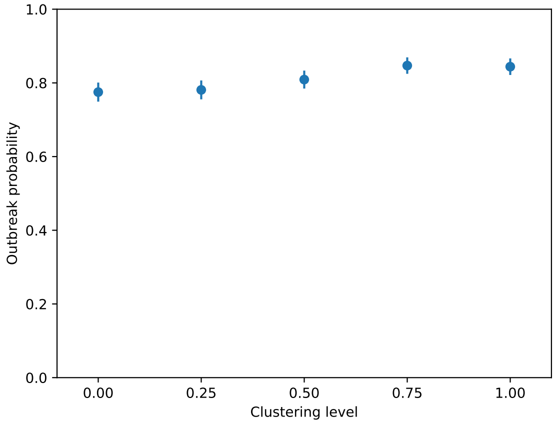

(c) Transmission probability = 0.70 ($\hat{R_0} = 20.35$)

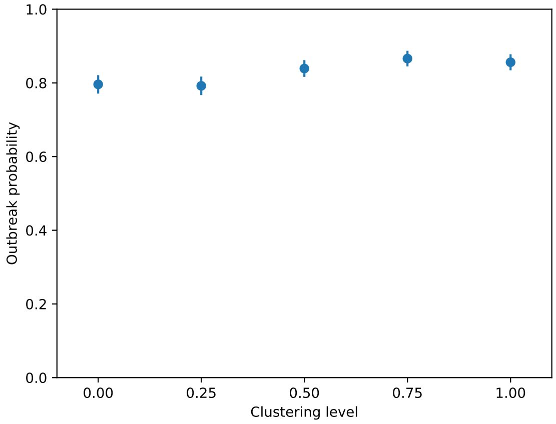

(d) Transmission probability = 0.75 ($\hat{R_0} = 21.48$)

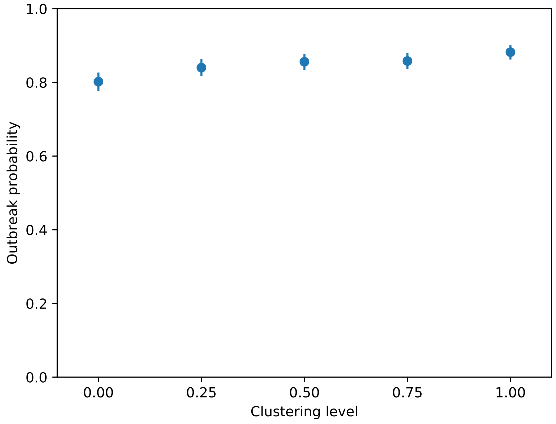

(e) Transmission probability = 0.80 ($\hat{R_0} = 22.58$)

Supplementary Figure S8. Probability of the occurrence of a persistent outbreak for clustering levels 0, 0.25, 0.5, 0.75, and 1 over transmission probabilities from 0.60 ($\hat{R_0} = 17.99$) to 0.80 ($\hat{R_0} = 22.58$). Results over 1,000 stochastic simulations per combination of transmission probability and clustering level. Error bars indicate 95% confidence intervals, for scenarios where enough (at least 5 of each) persistent outbreaks and extinction cases occurred.

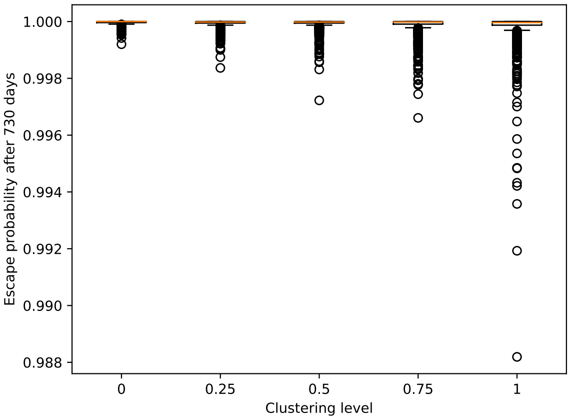

(a) Transmission probability = 0.20 ($\hat{R_0} = 6.80$)

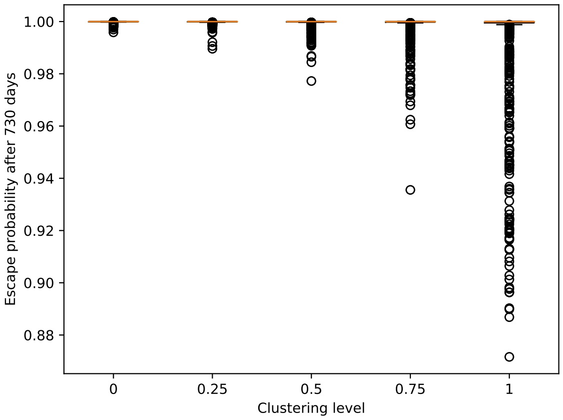

(b) Transmission probability = 0.25 ($\hat{R_0} = 8.38$)

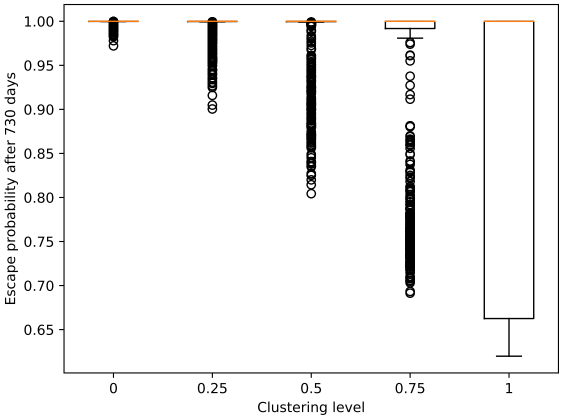

(c) Transmission probability = 0.30 ($\hat{R_0} = 9.91$)

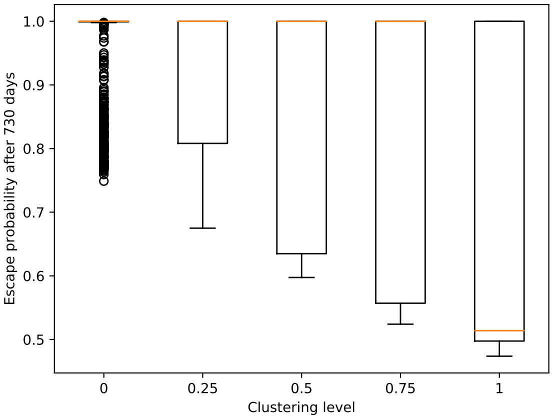

(d) Transmission probability = 0.35 ($\hat{R_0} = 11.38$)

Supplementary Figure S9. Box-plots of escape probabilities for clustering levels 0, 0.25, 0.5, 0.75, and 1 over transmission probabilities from 0.20 ($\hat{R_0} = 6.80$) to 0.35 ($\hat{R_0} = 11.38$). Results over 1,000 stochastic simulations per combination of transmission probability and clustering level.

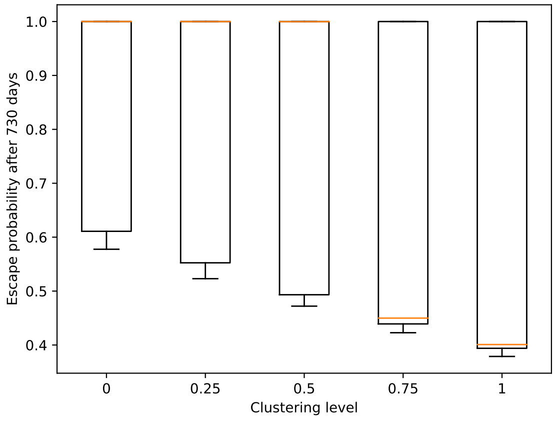

(a) Transmission probability = 0.40 ($\hat{R}_0 = 12.80$)

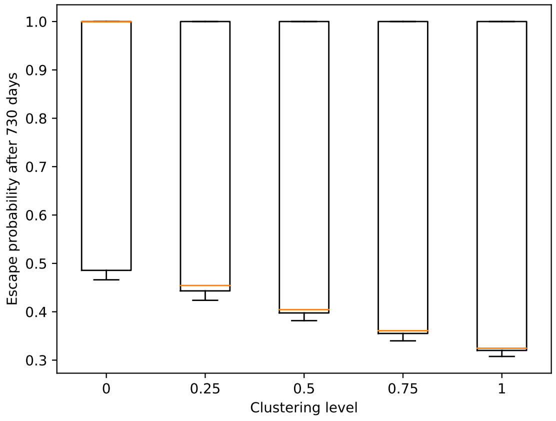

(b) Transmission probability = 0.45 ($\hat{R}_0 = 14.16$)

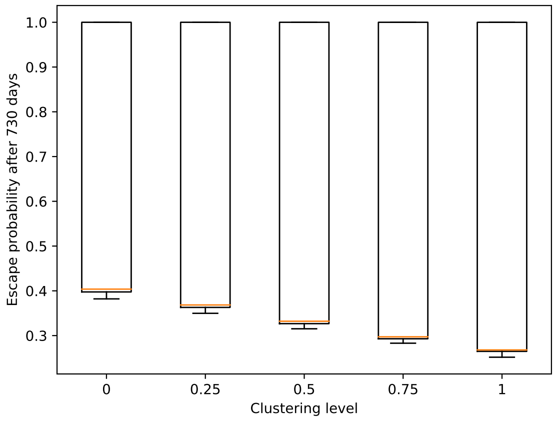

(c) Transmission probability = 0.50 ($\hat{R}_0 = 15.48$)

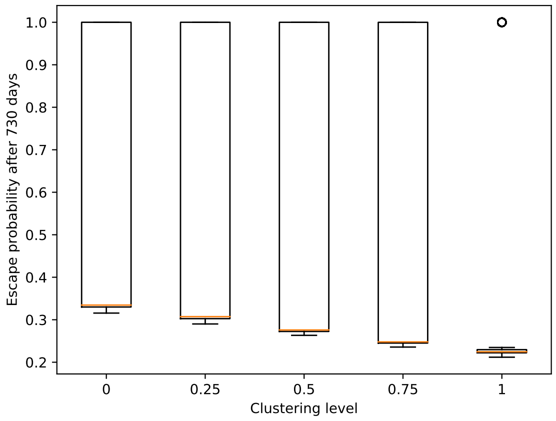

(d) Transmission probability = 0.55 ($\hat{R}_0 = 16.76$)

Supplementary Figure S10. Box-plots of escape probabilities for clustering levels 0, 0.25, 0.5, 0.75, and 1 over transmission probabilities from 0.40 ($\hat{R}_0 = 12.80$) to 0.55 ($\hat{R}_0 = 16.76$). Results over 1,000 stochastic simulations per combination of transmission probability and clustering level.

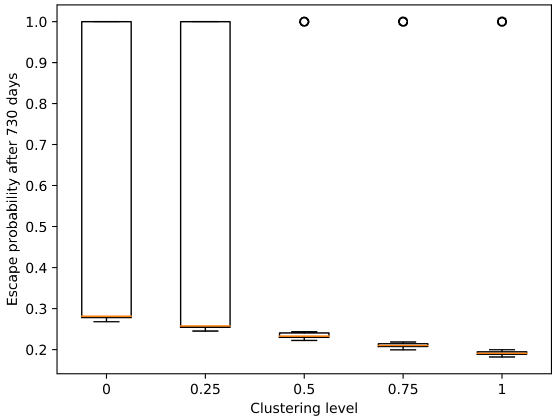

(a) Transmission probability = 0.60 ($\hat{R}_0 = 17.99$)

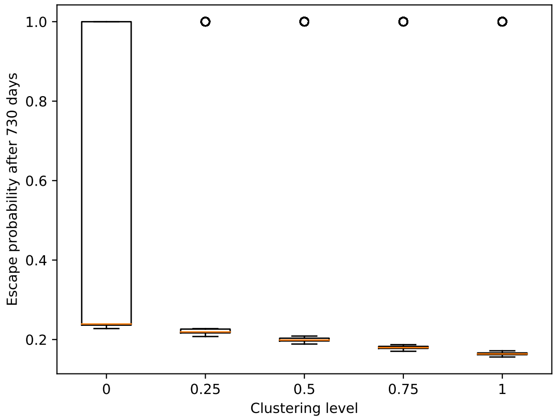

(b) Transmission probability = 0.65 ($\hat{R}_0 = 19.19$)

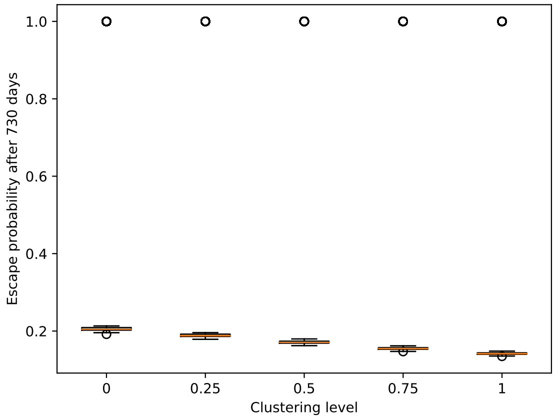

(c) Transmission probability = 0.70 ($\hat{R}_0 = 20.35$)

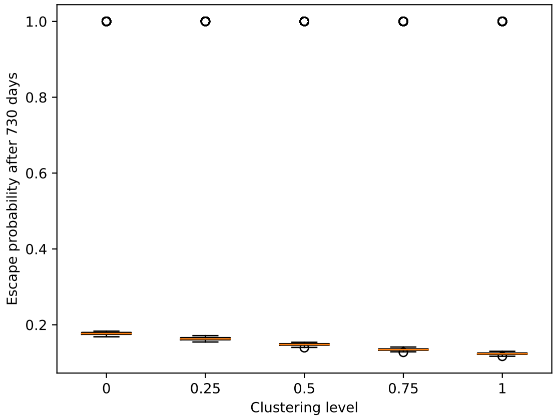

(d) Transmission probability = 0.75 ($\hat{R}_0 = 21.48$)

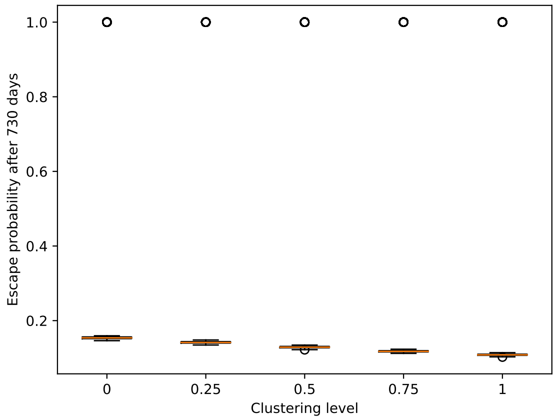

(e) Transmission probability = 0.80 ($\hat{R}_0 = 22.58$)

Supplementary Figure S11. Box-plots of escape probabilities for clustering levels 0, 0.25, 0.5, 0.75, and 1 over transmission probabilities from 0.60 ($\hat{R}_0 = 17.99$) to 0.80 ($\hat{R}_0 = 22.58$). Results over 1,000 stochastic simulations per combination of transmission probability and clustering level.

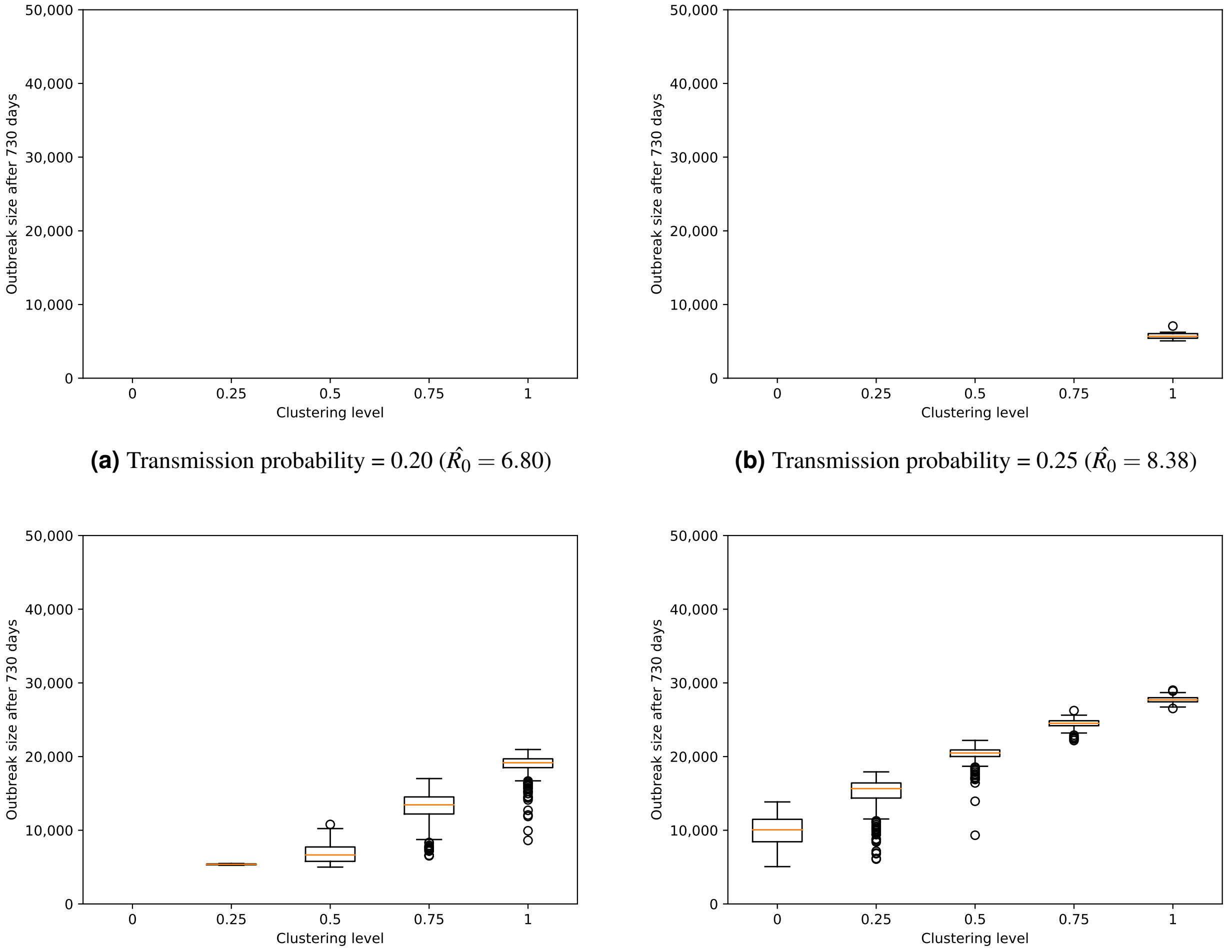

(a) Transmission probability = 0.20 ($\hat{R_0} = 6.80$)

(b) Transmission probability = 0.25 ($\hat{R_0} = 8.38$)

(c) Transmission probability = 0.30 ($\hat{R_0} = 9.91$)

(d) Transmission probability = 0.35 ($\hat{R_0} = 11.38$)

Supplementary Figure S12. Box-plots of outbreak sizes of persistent outbreaks (extinction threshold = 5,000 cases) for clustering levels 0, 0.25, 0.5, 0.75, and 1 over transmission probabilities from 0.20 ($\hat{R_0} = 6.80$) to 0.35 ($\hat{R_0} = 11.38$). Results over 1,000 stochastic simulations per combination of transmission probability and clustering level.

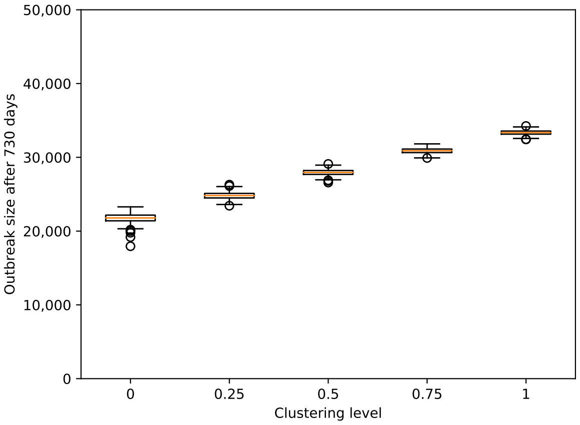

(a) Transmission probability = 0.40 ($\hat{R_0} = 12.80$)

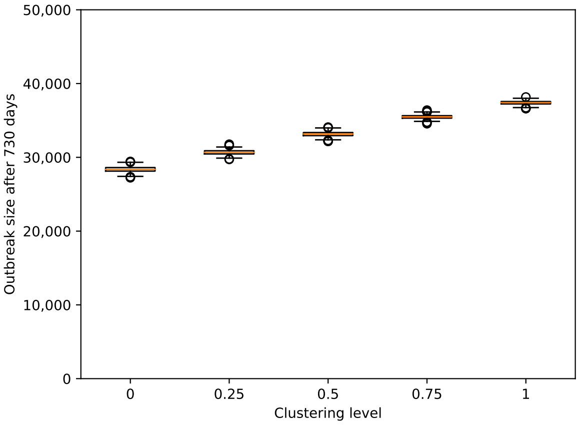

(b) Transmission probability = 0.45 ($\hat{R_0} = 14.16$)

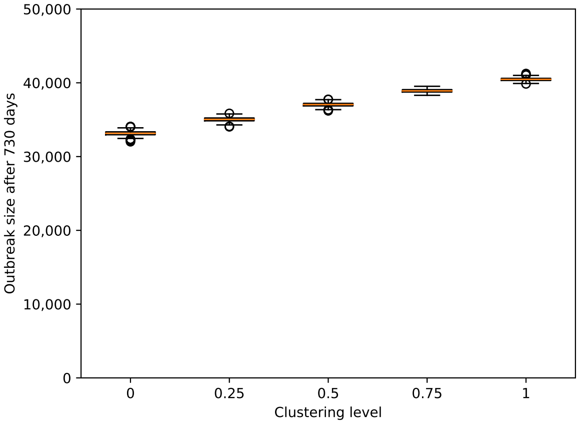

(c) Transmission probability = 0.50 ($\hat{R_0} = 15.48$)

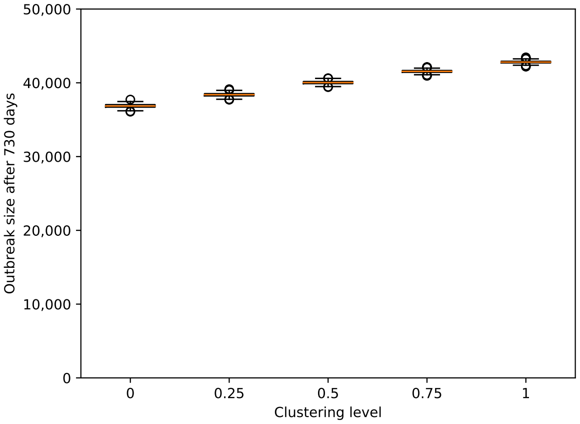

(d) Transmission probability = 0.55 ($\hat{R_0} = 16.76$)

Supplementary Figure S13. Box-plots of outbreak sizes of persistent outbreaks (extinction threshold = 5,000 cases) for clustering levels 0, 0.25, 0.5, 0.75, and 1 over transmission probabilities from 0.40 ($\hat{R_0} = 12.80$) to 0.55 ($\hat{R_0} = 16.76$). Results over 1,000 stochastic simulations per combination of transmission probability and clustering level.

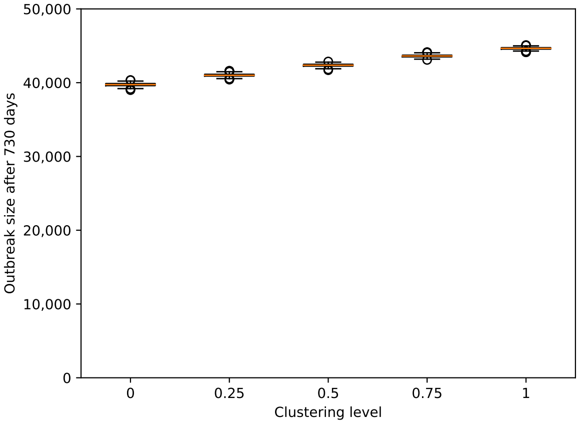

(a) Transmission probability = 0.60 ($\hat{R}_0 = 17.99$)

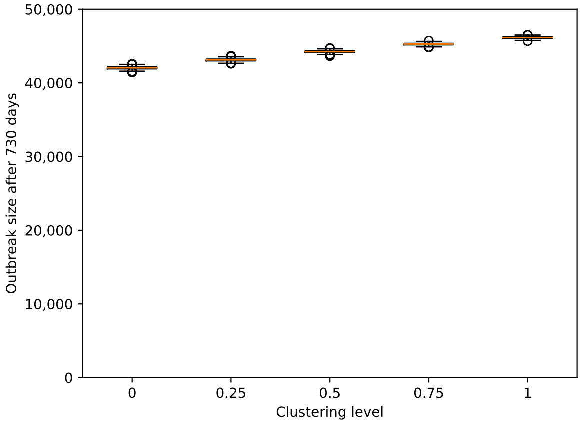

(b) Transmission probability = 0.65 ($\hat{R}_0 = 19.19$)

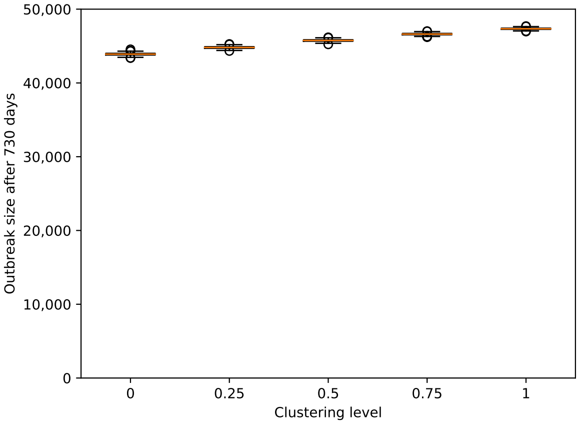

(c) Transmission probability = 0.70 ($\hat{R}_0 = 20.35$)

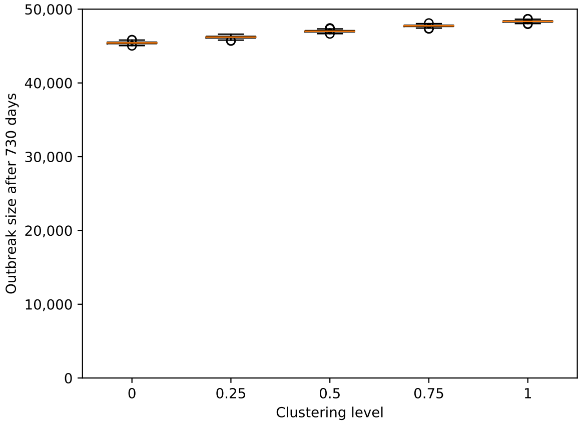

(d) Transmission probability = 0.75 ($\hat{R}_0 = 21.48$)

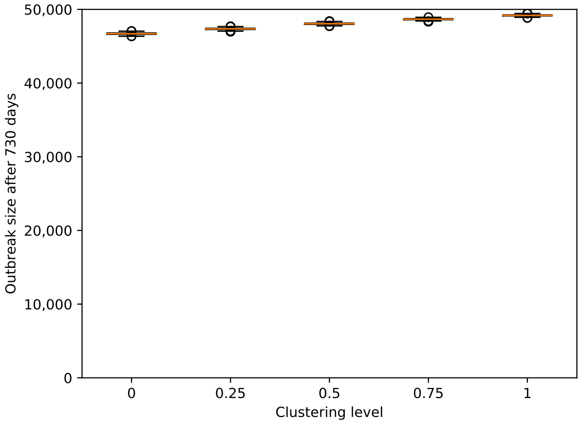

(e) Transmission probability = 0.80 ($\hat{R}_0 = 22.58$)

Supplementary Figure S14. Box-plots of outbreak sizes of persistent outbreaks (extinction threshold = 5,000 cases) for clustering levels 0, 0.25, 0.5, 0.75, and 1 over transmission probabilities from 0.60 ($\hat{R}_0 = 17.99$) to 0.80 ($\hat{R}_0 = 22.58$). Results over 1,000 stochastic simulations per combination of transmission probability and clustering level.

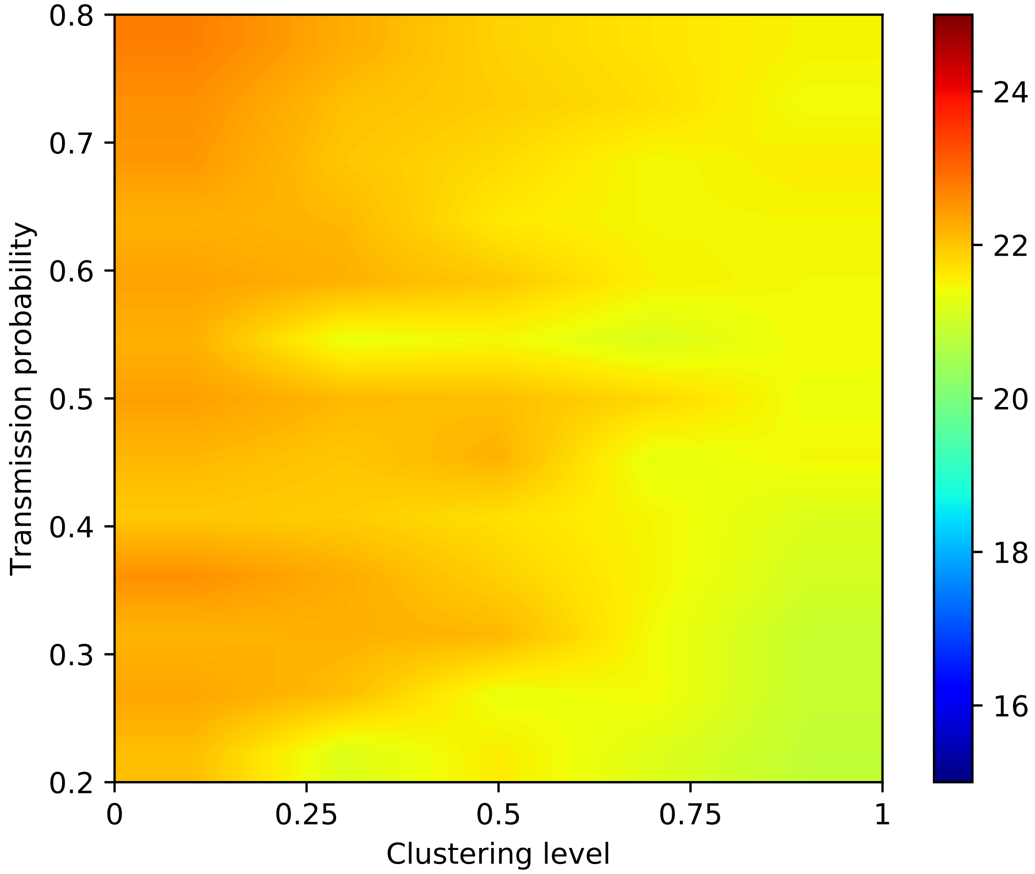

Supplementary Figure S15. Average ages of infected individuals for clustering levels 0 to 1 and transmission probabilities 0.2 to 0.8 ($R_0 \sim 6.80$ to 22.58). We ran 1,000 simulations for each scenario and calculated the average age over all infected individuals.
